# Rigid Posterior Lumbopelvic Fixation without Formal Debridement for Pyogenic Vertebral Diskitis and Osteomyelitis Involving the Lumbosacral Junction: Technical Report

**DOI:** 10.3389/fsurg.2015.00047

**Published:** 2015-09-22

**Authors:** Marcus D. Mazur, Vijay M. Ravindra, Andrew T. Dailey, Sara McEvoy, Meic H. Schmidt

**Affiliations:** ^1^Department of Neurosurgery, Clinical Neurosciences Center, University of Utah, Salt Lake City, UT, USA

**Keywords:** diskitis, osteomyelitis, S2-alar-iliac screw, lumbosacral, pyogenic, posterior, pelvic fixation

## Abstract

**Background:**

Pelvic fixation with S2-alar-iliac (S2AI) screws can increase the rigidity of a lumbosacral construct, which may promote bone healing, improve antibiotic delivery to infected tissues, and avoid L5–S1 pseudarthrosis.

**Purpose:**

To describe the use of single-stage posterior fixation without debridement for the treatment of pyogenic vertebral diskitis and osteomyelitis (PVDO) at the lumbosacral junction.

**Study design:**

Technical report.

**Methods:**

We describe the management of PVDO at the lumbosacral junction in which the infection invaded the endplates, disk space, vertebrae, prevertebral soft tissues, and epidural space. Pedicle involvement precluded screw fixation at L5. Surgical management consisted of a single-stage posterior operation with rigid lumbopelvic fixation augmented with S2-alar-iliac screws and without formal debridement of the infected area, followed by long-term antibiotic treatment.

**Results:**

At 2-year follow-up, successful fusion and eradication of the infection were achieved.

**Conclusion:**

PVDO at the lumbosacral junction may be treated successfully using rigid posterior-only fixation without formal debridement combined with antibiotic therapy.

## Introduction

Although antibiotics are the mainstay of therapy for pyogenic vertebral diskitis osteomyelitis (PVDO), surgery may be necessary for patients with neurological impairment, spinal instability, and failure of antibiotic therapy. A few reports have described the management of PVDO using posterior-only fixation without formal debridement of the involved vertebrae, disks, and surrounding soft tissues (1–3). We report the technique we have used for treatment of PVDO at the lumbosacral junction using pelvic fixation bolstered with S2-alar-iliac (S2AI) screws, without formal debridement of the involved anterior column, combined with antibiotic therapy. Two patients have been successfully treated using this technique.

## Case Illustration

A 67-year-old diabetic woman presented with severe back pain from PVDO involving L4–L5–S1 that had originated from a sacral decubitus ulcer. Her pain persisted despite 2 months of broad-spectrum antibiotic therapy. On admission to our hospital, her laboratory results indicated a white blood cell count (WBC) of 6.6 × 10^3^/μL, an erythrocyte sedimentation rate (ESR) of 68 mm/h, and a C-reactive protein (CRP) concentration of 54.5 mg/L. A computed tomography (CT)-guided L5 pedicle biopsy grew vancomycin-resistant *Enterococcus*, *Klebsiella*, methicillin-resistant *Staphylococcus aureus*, and *Candida albicans*. The patient was treated with daptomycin, meropenem, and fluconazole.

**Figure 1:**
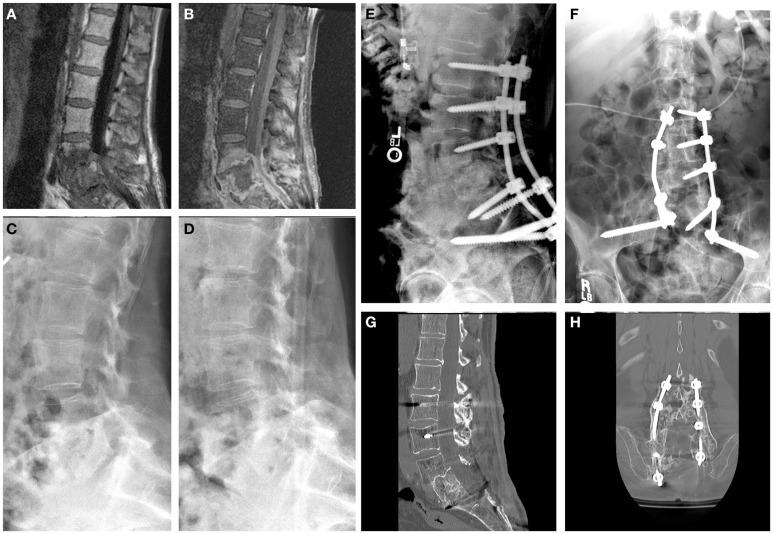
**Patient 1**. A 67-year-old woman with severe back pain presented after medical treatment of a polymicrobial sacral decubitus ulcer. Sagittal T1-weighted magnetic resonance images of the lumbar spine with **(A)** and without **(B)** contrast demonstrated PVDO at L4–L5–S1 with involvement of the vertebral bodies and endplates. L4–L5 spondylodiscitis with anterolisthesis was present as well as epidural and prevertebral abscesses. Preoperative upright radiographs before **(C)** and 1 month after **(D)** antibiotic therapy demonstrate progression from Grade I to Grade II L4–L5 anterolisthesis. Posterior-only decompression and fusion without formal debridement of the infected tissues was performed with pedicle screws at L2, L3, right L4, and S1 combined with S2AI screws **(E,F)**; poor bone quality precluded instrumentation at L4 on the left and at L5. At 2 years, sagittal **(G)** and coronal **(H)** CT reconstructions demonstrate stable fusion at the lumbosacral junction.

Imaging demonstrated PVDO of L4–L5–S1 with endplate erosion, L4–L5 spondylodiscitis with anterolisthesis, and collapse of the L4–L5 and L5–S1 disk spaces (Figure 1). The infection extended into the epidural and prevertebral regions, and there was progression of L4–L5 anterolisthesis despite medical treatment.

### Operation

Because the patient had mechanical back pain with anterolisthesis, instability was suspected so a single-stage decompression, posterior instrumentation, and fusion from L2 to the ilium were performed using standard open technique. A midline incision was made 2 cm rostral to the sacral decubitus. Laminectomies were performed at L4, L5, and S1. Because the infection had eroded the endplates and obliterated the disk spaces of L4, L5, and S1, interbody grafts were not placed to avoid subsidence and further instability. The patient had adequate lumbar lordosis, so the decision was made to fuse *in situ* via a posterior-only approach.

All screws were inserted using O-arm Surgical Imaging System/StealthStation imaging guidance. Pelvic fixation was achieved using 8.5 mm × 85 mm (right) and 8.5 mm × 95 mm (left) S2AI screws. Screws could not be inserted into the right pedicle of L4 or any portion of L5, so the construct consisted of instrumentation at L2, L3, left L4, S1, and the pelvis. The S2AI screws were aligned with rostral instrumentation without the use of connector devices. The rod–screw construct was assembled, and 150 mL of morselized allograft was placed in the lateral gutters with 11.2 cm^3^ of recombinant bone morphogenic protein.

The 4 cm × 3 cm sacral decubitus ulcer had a clean base with granulation tissue and no purulent material. Thus, a definitive flap closure of the ulcer was not performed for fear of contaminating the lumbar wound and spinal instrumentation. Rather, the lumbar incision was closed separately with fasciomusculocutaneous advancement flaps, the ulcer was debrided with a curette, and a vacuum-assisted closure (VAC) device was placed on the infected area.

The patient was managed without a brace. Two weeks after the operation, her WBC, ESR, and CRP decreased to 3.9 × 10^3^/μL, 53 mm/h, and 2.6 mg/L, respectively. She continued an 8-week course of daptomycin, meropenem, and fluconazole, followed by suppressive therapy with oral doxycycline. The sacral ulcer healed by secondary intention after several weeks of wound VAC therapy. A CT scan at 2-year follow-up showed excellent fusion between L2 and the sacrum and no evidence of loosening or backout of the pedicle or S2AI screws. Had the patient developed worsening pain, new neurological deficit, deformity progression, or symptomatic pseudarthrosis, we would have considered performing anterior stabilization as a second operation after the infection was treated. She was able to walk short distances with a front-wheeled walker.

## Discussion

**Figure 2:**
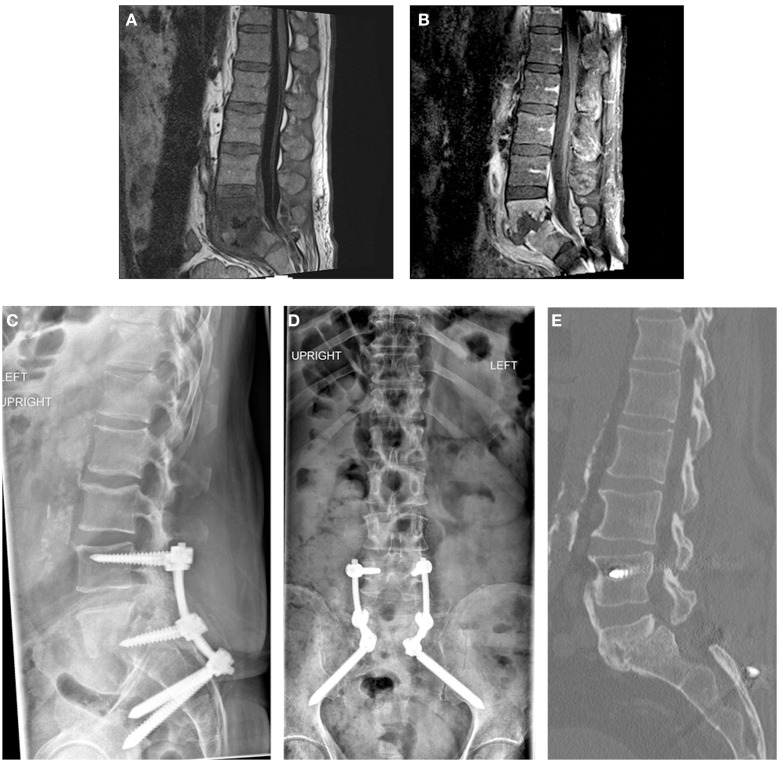
**Patient 2**. A 56-year-old man presented with mechanical low back pain. Sagittal T1-weighted magnetic resonance images of the lumbar spine with **(A)** and without **(B)** contrast demonstrated PVDO at L5–S1 with erosion of the endplates and collapse of the disk space. Epidural and prevertebral abscesses were also present. Posterior-only decompression and posterolateral fusion without formal debridement of the L5–S1 disk space was performed. Intraoperative cultures failed to yield a causative organism. Posterior instrumentation included pedicle screws at L4 and S1 with S2AI screws **(C,D)**; poor bone quality precluded instrumentation of L5. Broad-spectrum antibiotics with vancomycin and ceftriaxone were administered for 6 weeks, followed by oral doxycycline suppressive therapy for 2 years. At 2 years, CT demonstrated a solid fusion anteriorly between L5 and S1 **(E)**; bridging bone is also seen between L4 and L5.

Rigid fixation combined with antibiotics can effectively treat PVDO at the lumbosacral junction without formal debridement of the infected tissues. Only a few reports have described outcomes in patients in whom posterior approaches for PVDO were performed without entering the disk space and without removing the infected bone (1–3). Appropriate antibiotic treatment is mandatory. We have observed good results after obtaining cultures and initiating preoperative targeted antibiotic therapy. Nonetheless, prolonged antimicrobial treatment is required for an optimal long-term outcome (4). Instrumentation may also be necessary to prevent deformity, particularly for PVDO at a mobile segment of the spine, such as the lumbosacral junction. Rigid fixation stabilizes the site to promote bone healing by improving blood flow to the disrupted vertebrae, allowing better penetration of antibiotics to the infected tissues (3, 5). In this report, we describe how pelvic fixation with S2AI screws can bolster construct rigidity to enable fusion in the setting of PVDO even when pedicle screw fixation at L5 cannot be achieved. Two patients have been treated successfully in this manner at our institution, and both achieved a solid fusion with eradication of the infection at 2-year follow-up (Figures 1 and 2).

Pelvic fixation increases the rigidity of instrumentation and reduces strain on S1 screws, preventing S1 screw loosening and lumbosacral pseudoarthrosis (6). S2AI screws have several potential advantages over iliac bolts: greater biomechanical pullout strength because S2AI screws cross the cortical bone at the dorsal sacrum and sacroiliac joint and are anchored in the thick bone above the greater sciatic notch (7); lower profile because the insertion site of S2AI screws is located deeper than a traditional iliac bolt, thus enabling more muscle and fascial coverage of the instrumentation (8); and a midline incision site that allows alignment with the rostral lumbar instrumentation and connecting rod, thereby obviating the need for bulky connector devices.

S2AI screws are associated with fewer reoperations than iliac bolts (9). This is particularly relevant in patients with PVDO, who typically have comorbidities, such as diabetes (as seen in both of our patients), and are prone to poor wound healing and recurrent infections (4, 10). Hardware prominence is an important consideration in this population, which is prone to wound breakdown. We avoided the bulky prominence of connector devices because we used S2AI screws in our patients.

In an ideal setting, anterior column support combined with posterior fixation is preferred to remove the infection and stabilize the spine. But this approach may not be practical for patients who cannot tolerate the potential morbidity from an anterior or combined approach. Our case shows that a single-stage posterior-only decompression and instrumentation combined with antibiotics is a reasonable first option for patients who are at increased preoperative risk of complications. Nonetheless, patients should be managed expectantly and anterior stabilization may be necessary if symptomatic pseudarthrosis, worsening pain or neurological deficit, recurrent infection, or deformity progression occurs.

## Informed consent

Both patients in this report have granted written informed consent to include their images and clinical information.

## Conflict of Interest Statement

The authors declare that the research was conducted in the absence of any commercial or financial relationships that could be construed as a potential conflict of interest.

## Abbreviations

CRP, C-reactive protein; CT, computed tomography; ESR, erythrocyte sedimentation rate; MR, magnetic resonance; MRSA, methicillin-resistant *Staphylococcus aureus*; S2AI, S2-alar-iliac; WBC, white blood cell count.
